# DiffHiChIP: Identifying differential chromatin contacts from HiChIP data

**DOI:** 10.1016/j.crmeth.2025.101214

**Published:** 2025-11-03

**Authors:** Sourya Bhattacharyya, Daniela Salgado Figueroa, Katia Georgopoulos, Ferhat Ay

**Affiliations:** 1 La Jolla Institute for Immunology, La Jolla, CA 92037, USA; 2 Bioinformatics and Systems Biology Program, University of California, San Diego, La Jolla, CA 92093, USA; 3 Cutaneous Biology Research Center, Massachusetts General Hospital, Harvard Medical School, Charlestown, MA 02129, USA; 4 Department of Pediatrics, University of California, San Diego, La Jolla, CA 92093, USA

**Keywords:** 3D genome organization, HiChIP, differential loops, chromatin looping, differential analysis

## Abstract

Chromosome conformation capture (3C) assays such as HiChIP are widely used to study interactions between *cis*-regulatory and structural elements. However, robust methods for detecting condition-specific loops remain limited. We introduce DiffHiChIP, the first comprehensive framework to call differential loops from HiChIP and similar 3C protocols. DiffHiChIP supports DESeq2 and edgeR using either a complete contact map or a subset of contacts for background estimation, incorporates edgeR with generalized linear model (GLM) using either quasi-likelihood F test or likelihood ratio test, and implements independent hypothesis weighting (IHW) as well as a distance stratification technique for modeling distance decay of contacts in estimating statistical significance. Our results on five datasets suggest that edgeR GLM-based models with IHW correction reliably capture differential interactions, including long-range interactions, that are supported by published Hi-C data and reference studies. As HiChIP data become increasingly used for modeling chromatin regulation, DiffHiChIP promises to have a broad impact and utility.

## Highlights

• DiffHiChIP is a comprehensive framework to detect differential HiChIP loops
• Captures long-range differential loops by modeling contact distance decay
• Highlights differences in sensitivity and specificity of utilized statistical tests
• Benchmarking across five datasets guides optimal settings for differential analysis

## Motivation

High-resolution conformation capture assays such as HiChIP are commonly used for profiling chromatin loops. Detection of differential loops between two conditions (e.g., same cell type, different genotypes, or before/after perturbations) helps contextualize condition-specific activities of genes in connection with such *cis*-regulatory elements. However, existing differential loop callers for HiChIP data employ count-based models that are designed with gene expression data in mind and, hence, do not account for the distance decay of contact counts from HiChIP data. These approaches are not ideal for the detection of differential long-range (>400 kb) loops, a limitation that persists even with the use of implicit or explicit corrections for this distance effect. This highlights the need for a specialized framework that addresses the unique properties of HiChIP data to enable accurate and robust detection of differential chromatin interactions.

## Graphical abstract

**Figure:**
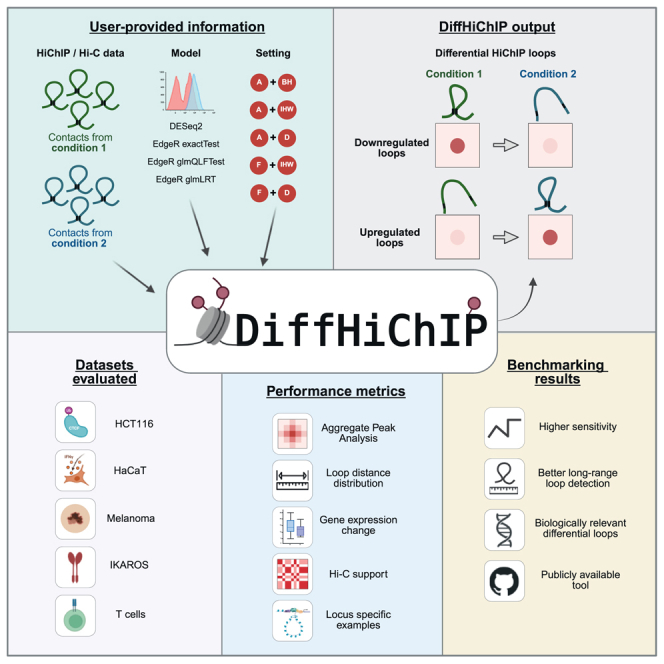

## Teaser

Bhattacharyya et al. present DiffHiChIP, a statistical framework for detecting differential chromatin loops from HiChIP data. By modeling distance decay of contacts and integrating multiple statistical models and dispersion estimation strategies, DiffHiChIP improves detection of long-range differential interactions and provides a robust tool for studying condition-specific chromatin regulation.

## Introduction

Chromosome conformation capture (3C) technologies such as Hi-C^1^^,^^2^ and its variants such as promoter capture Hi-C (PCHi-C),^3^^,^^4^ ChIA-PET,^5^ Micro-C,^6^ and others produce high-resolution 3D chromatin interaction maps of the genome.^7^ One variant, commonly referred to as HiChIP (Hi-C coupled with chromatin immunoprecipitation or proximity ligation assisted chromatin immunoprecipitation sequencing [ChIP-seq])^8^^,^^9^^,^^10^ profiles protein- or histone modification-centric (e.g., CTCF or H3K27ac) interactions requiring much lower sequencing depth than Hi-C (∼300 M HiChIP reads instead of >1 B Hi-C reads for 5 kb resolution). This allows more scalable and cost-effective studies of genome-wide chromatin interactions or loops (we reserve the term loop for interactions that meet certain significance criteria) across regulatory and/or structural elements from multiple different cell types and conditions.^11^^,^^12^^,^^13^^,^^14^^,^^15^ The increasing availability of HiChIP datasets also prompts the need to systematically identify their similarities and differences across conditions. Although various methods have assessed the reproducibility^16^^,^^17^^,^^18^ and differential loops^19^^,^^20^^,^^21^^,^^22^ from Hi-C and PCHi-C contact maps, similar studies for HiChIP data are limited.^23^^,^^24^^,^^25^^,^^26^ This gap is partly due to the challenges of modeling the non-uniform coverage of HiChIP contact counts according to the underlying ChIP-seq (1D) signal and the genomic distance between interacting loci. Existing differential HiChIP callers such as diffloop,^25^ HiCDC+,^24^ and FitHiChIP^23^ have employed RNA sequencing (RNA-seq) count-based techniques DESeq2^27^ and edgeR.^28^ While DESeq2 employs negative binomial distribution on the input count matrix and generalized linear model (GLM)-based regression to estimate the gene-wise dispersions, edgeR exactTest uses the percentiles (or rank) of gene expression to compute gene-wise dispersions and estimate gene-specific *p* values using the quantile normalized RNA-seq counts.^29^ However, HiChIP contacts exhibit much higher dispersion than 1D RNA-seq and ChIP-seq assays due to inherent 3C-based biases (e.g., position of restriction fragments, and distance effect) as well as underlying ChIP-seq signals.^30^^,^^31^ We reasoned that GLM-based regression in edgeR employing both gene-wise and common (or trended) dispersion together with likelihood ratio test (LRT)^29^ or quasi-likelihood-F test (QLFTest)^32^ would likely fit the HiChIP contacts better than the exactTest setting, akin to their application in estimating differential abundance of single-cell clusters.^33^^,^^34^ Additionally, neither DESeq2 nor edgeR models the exponential distance decay of chromatin contacts,^35^^,^^36^ thus mostly ignoring the longer-range (>400 kb) differential loops. To model such distance decay in estimating the *p* values of chromatin contacts, previous studies have either employed independent hypothesis weighting (IHW)^37^ for false discovery rate (FDR) correction on normalized Hi-C contact maps^38^ or applied distance stratification to first distribute the chromatin contacts into different bins subject to their genomic distances and then estimate statistical significance separately for individual bins.^24^^,^^39^ Relative utilities of these distance decay modeling techniques remain to be systematically benchmarked with comprehensive datasets and metrics that utilize orthogonal data to assess their accuracy.

Here, we present DiffHiChIP, a comprehensive framework to identify differential HiChIP loops by integrating various count-based approaches (DESeq2 or edgeR), supporting multiple dispersion estimation techniques (exactTest or GLM), employing different statistical tests (LRT and QLFTest), and modeling distance decay in multiple ways (e.g., IHW and distance stratification). We provide a comprehensive assessment of each of these aspects of differential HiChIP analysis, including comparisons to previously published methods (Table 1), using 5 different HiChIP datasets spanning perturbations of regulators of chromatin looping, cytokine stimulation, and different cell types. We also utilize matched data from Hi-C, ChIP-seq, and RNA-seq experiments in these conditions as well as gene/loci highlighted in these studies for evaluating differential loop calls. Our results suggest that (1) IHW correction of *p* values generally performs better in capturing longer-range differential HiChIP loops compared to BH correction or distance stratification; (2) GLM-based statistical tests in edgeR exhibit higher sensitivity of differential loop calling than DESeq2 and edgeR exactTest models, particularly for datasets with lower number of replicates (*n* = 2), as well as previously published HiCDC+ model; and (3) for datasets with higher number of replicates per condition, DESeq2 reports considerably higher number of differential loops but with lower specificity. Although the results vary substantially across different HiChIP datasets for some of these metrics, our findings point to specific settings and statistical parameters to improve differential HiChIP analysis. DiffHiChIP is publicly available at https://github.com/ay-lab/DiffHiChIP.

**Table 1:** Comparison of count-based methods, dispersion estimation techniques, statistical tests, distance decay models, and differential 1D bins approaches used by DiffHiChIP (this work) and other published work

General feature	Specific feature	DiffHiChIP	HiCDC+ (Sahin et al., 2021^24^)	FitHiChIP (Bhattacharyya et al., 2019^23^)	diffloop (Lareau et al., 2018^25^)	Gorkin et al., 2019^38^	Kubo et al., 2021^39^
Count-based method	DEseq2	⊠	⊠	☐	☐	☐	☐
	EdgeR	⊠	☐	⊠	⊠	☐	⊠
	Limma	☐	☐	☐	⊠	☐	☐
Dispersion estimation technique	exactTest	⊠	☐	⊠	☐	N/A	N/S
	GLM	⊠	⊠	☐	⊠	N/A	N/S
Statistical tests	LRT	⊠	N/A	☐	⊠	⊠	N/S
	QLFTest	⊠	N/A	☐	☐	☐	N/S
	Wald	☐	⊠	☐	☐	☐	N/S
Distance decay modeling	IHW [37]	⊠	☐	☐	☐	⊠	☐
	distance stratification	⊠	⊠	☐	☐	☐	⊠
Differential 1D bins	ChIP-seq overlap	⊠	☐	⊠	☐	N/A	⊠

N/A, not applicable; N/S, not specified.

## Results

### DiffHiChIP: A comprehensive framework for detecting differential loops from HiChIP data

**Figure 1:**
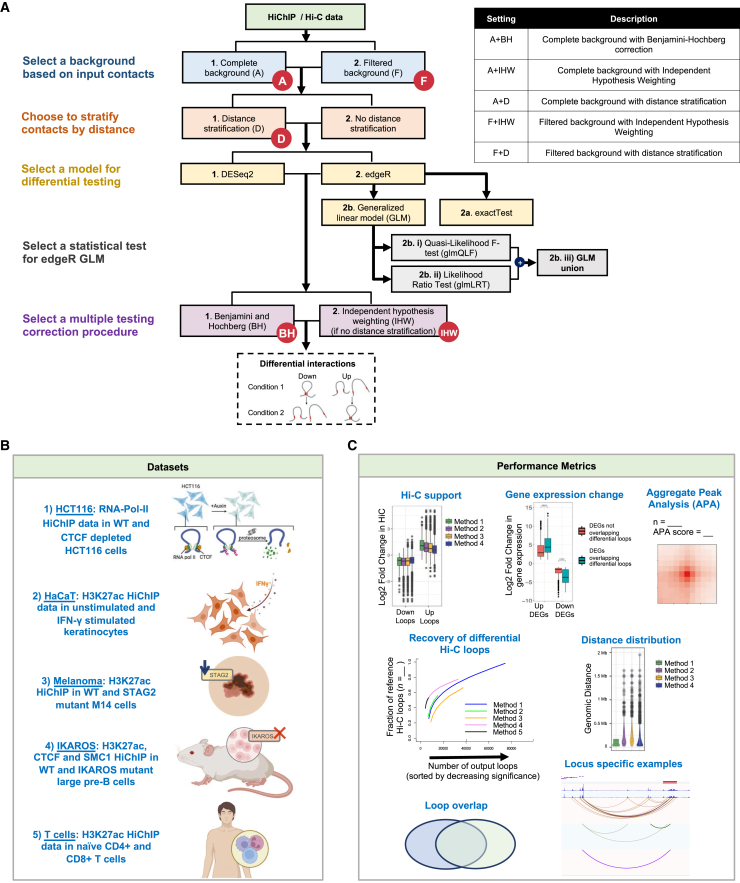
Workflow of DiffHiChIP (A) DiffHiChIP calls differential loops from HiChIP data and supports various configurations summarized in the table (right). (B) Schematic of the five different datasets employed in this study for performance validation (IKAROS has three different sets of HiChIP experiments). (C) Metrics employed to evaluate the differential loop calls reported by various settings of DiffHiChIP. Schematics in (B) were created with Biorender.com/.

DiffHiChIP calls differential chromatin loops/interactions/contacts from HiChIP data between two conditions (e.g., disease vs. control or between two different cell types) having one or more replicates (Figure 1A). DiffHiChIP supports both DESeq2 and edgeR as the underlying models for differential analysis. Specifically, for edgeR, DiffHiChIP includes both exactTest and GLM-based settings applied with different statistical tests (LRT or QLFTest). DiffHiChIP supports both Benjamini-Hochberg (denoted by BH in this manuscript) adjustment and independent hypothesis weighting (denoted as IHW in this work) to perform the multiple hypothesis testing correction of *p* values and FDR control. For IHW, DiffHiChIP uses either the mean normalized counts across conditions *(*baseMean, recommended by Love et al. and Ignatiadis et al.^27^^,^^37^) for DESeq2 or log counts per million (logCPM) for edgeR, as the independent covariates. DiffHiChIP also implements a custom distance stratification using equal occupancy binning (STAR Methods), inspired by FitHiC,^35^^,^^36^ and provides a comprehensive comparison between these distance decay modeling techniques (Figure 1A). As both DESeq2 and edgeR rely on background count distributions for statistical modeling, DiffHiChIP further supports two different settings of background contacts for these models (Figure 1A). The first setting, denoted as the complete background or A for all, uses the union of nonzero HiChIP contacts (contact count >0, significant or not) from all input samples. The second setting, denoted as the filtered background or F, employs the HiChIP contacts significant in at least one input sample, according to a user-defined FDR threshold *t* (default 0.1) for FitHiChIP^23^ calls. DiffHiChIP provides a comparative assessment between these background settings. If the custom distance stratification with equal occupancy binning is employed, corresponding settings are denoted by A + D and F + D for the complete and filtered backgrounds, respectively.

#### Description of datasets used for assessment of differential loop calls

We assessed DiffHiChIP and its various settings using five HiChIP datasets (Figure 1B, STAR Methods): (1) HCT116: RNA-Pol-II HiChIP from HCT116 colorectal cancer cells^14^ between control and Auxin treatment (CTCF depletion) conditions; (2) HaCaT: H3K27ac HiChIP from keratinocytes (HaCaT) cells^15^ between wild-type (WT) and interferon (IFN)-γ stimulated (stim) conditions; (3) Melanoma: H3K27ac HiChIP from M14 melanoma cells^13^ between WT and STAG2 knockdown (STAG2-KD) conditions; (4) IKAROS: H3K27ac, CTCF, and SMC1 HiChIP datasets from large pre-B cells in two conditions, namely WT IKAROS and DNA-binding domain mutant IKAROS (IKDN)^40^; and (5) T cells: H3K27ac HiChIP data of naive CD4^+^ and CD8^+^ T cells from healthy blood donors.^11^ Datasets 1 to 4 have accompanying RNA-seq, Hi-C, and ChIP-seq data for the corresponding conditions (except for dataset 3—Melanoma, which lacks ChIP-seq), along with 2 HiChIP replicates per condition. The dataset 5, on the other hand, has 6 HiChIP replicates per condition. After preprocessing HiChIP datasets, we used FitHiChIP^23^ to call significant HiChIP loops (Data S1, STAR Methods) for different conditions and replicates and used them as the inputs for DiffHiChIP. Differential loops are then evaluated using metrics derived from the input HiChIP data and other matching data available from the same conditions (Figure 1C).

### Comparison of different distance stratification approaches

Previous works such as HiCDC+^24^ binned chromatin loops per 10 kb genomic distance and estimated DESeq2 size factors per bin, while another study^38^ computed the cumulative contact counts per 10 kb distance bins and compared with the contact counts of the interactions having 140–150 kb genomic distance. Both these techniques, however, did not capture long-range interactions. Thus, we implemented two new approaches to handle distance effect. The first approach adapts IHW by incorporating a covariate informative of the power of each test (ideally independent of *p* values) in FDR control of *p* values. We used the mean normalized counts (baseMean) for DESeq2 and logCPM for edgeR as the independent covariates for IHW correction. The second approach implements a custom distance stratification (setting D) by adapting the equal occupancy binning technique implemented in our earlier work.^23^^,^^36^ Here, chromatin contacts are stratified within a distance range (or bin) such that each bin would roughly have a similar number of contacts (STAR Methods). Each range is then used separately as input to the chosen model for statistical significance estimation followed by BH correction across all bins for FDR control.

**Figure 2:**
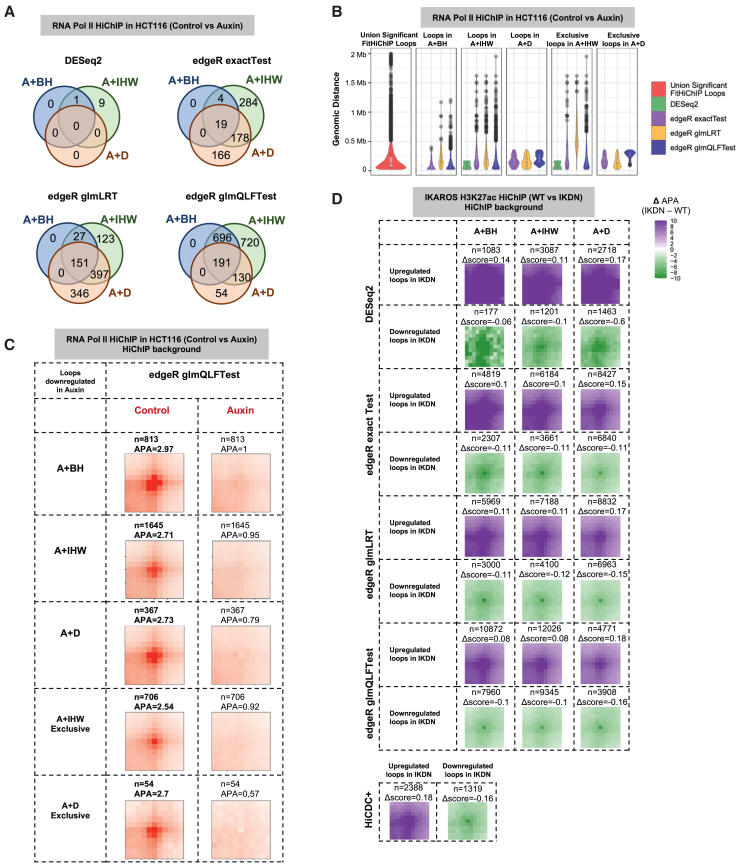
IHW correction detects longer-range differential loops better than BH or distance stratification (D) (A) Overlap of differential loops reported by A + BH, A + IHW, and A + D settings for various DESeq2 and edgeR settings using the HCT116 cell line Pol II HiChIP data from wild-type (control) and CTCF-depleted (Auxin) conditions. HiCDC+ detected zero differential loops (not shown). DiffHiChIP is executed in the complete background (A) setting. (B) Genomic distance distributions for either all or exclusive A + IHW or A + D differential loops, along with the union of significant FitHiChIP loops of all replicates, for the same dataset as (A). (C) Aggregate peak analysis (APA) plots for the differential loop categories reported in (B) using HiChIP data as background. The symbol *n* indicates the number of differential loops. (D) Differential APA plots (elementwise subtraction of the aggregate matrix for IKDN from that of WT) for IKAROS H3K27ac HiChIP data for various distance stratification settings and for HiCDC+. Differential APA scores (Δscore) represent the difference in APA scores between the IKDN and WT backgrounds.

Next, using the complete background (setting A) as our starting point, we evaluated the overlap of DiffHiChIP loops reported by three different settings: (1) BH correction of *p* values (A + BH) with no explicit distance correction, (2) IHW correction of *p* values (A + IHW) with contact count-based covariates, and (3) equal-occupancy-based distance stratification (A + D) before significance estimation. Across all datasets, differential loops reported by A + BH were mostly the subsets of the corresponding loops from A + IHW (Figures 2A and S1A–S1F). Numbers of loops exclusive to the setting A + IHW were generally higher than those exclusive to A + D setting, except when using the edgeR glmLRT model, where the opposite was observed (Figures 2A and S1A–S1F).

#### Capturing long-range differential loops by distance stratification

We next assessed the performance of the A + IHW and A + D settings in modeling the distance decay of chromatin contacts. Differential loops from A + IHW particularly with edgeR settings included a subset of long-range loops, similar to the overall set of significant loop calls, which were missing from A + D differential loops. For example, differential loops reported by the A + D setting were shorter range (distance <400 kb) for HCT116 data (Figure 2B). Across different datasets, the upper quartile (75^th^ percentile) of the loop distance distribution for loops exclusively detected by A + IHW was higher (260 kb–1.2 Mb) compared to those detected exclusively by A + D (90–660 kb) or by HiCDC+ (150–450 kb), suggesting that A + IHW captures longer-range differential loops more effectively (Figures 2B and S1G–S1L).

#### Support for differential loops identified by distance stratification from aggregate peak analysis of HiChIP data

We next employed aggregate peak analysis (APA)^2^^,^^23^ and differential APA (APA matrix of one condition subtracted from the other) to assess the relative enrichment of differential loops upregulated in specific conditions with respect to underlying HiChIP contact maps of the compared conditions (STAR Methods, Figure S2A), where higher magnitude of APA scores (or differential APA scores) indicates higher relative enrichment. Both the A + IHW and A + D settings, particularly when used with different edgeR configurations, reported similar APA (or differential APA) scores compared to the A + BH setting in spite of reporting a higher number of loops. In fact, loops exclusive to the A + IHW and A + D settings also showed high APA (or differential APA) scores across different datasets (Figures 2C, 2D, and S2B–S2H). In particular, loops exclusive to A + IHW setting showed some of the largest differential APA scores for IKAROS (pre-B cell) datasets between WT and DNA-binding mutant IKAROS (IKDN) conditions (Figures S2F–S2H). These results confirm the utility of IHW correction and distance stratification, compared to the classical BH adjustment of *p* values.

#### Support for differential loops identified by distance stratification from analysis of matched Hi-C data

**Figure 3:**
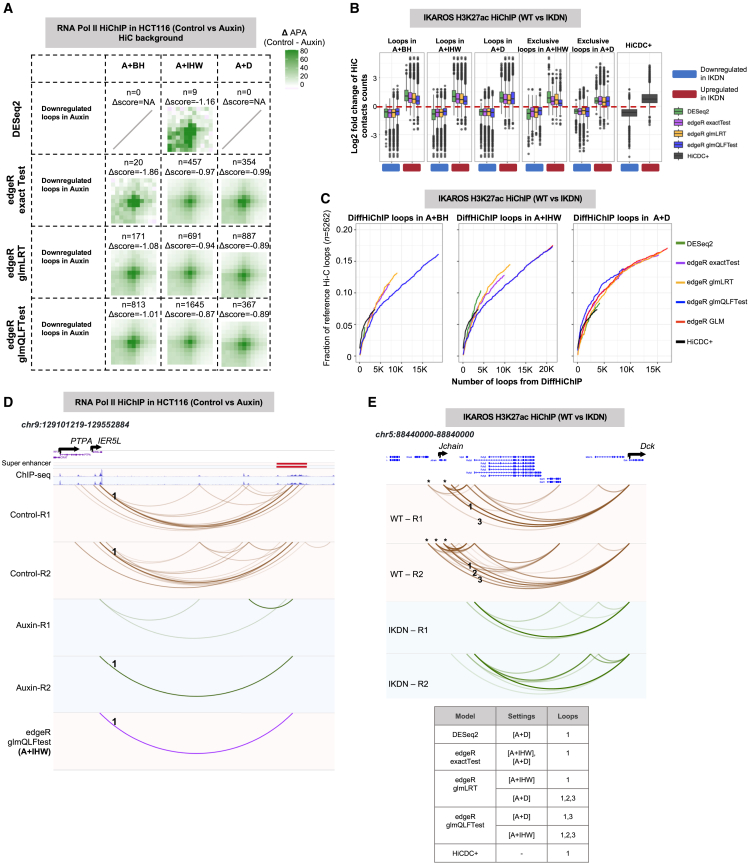
Further assessment of different distance stratification approaches (A) Differential APA plots for HCT116 cell line Pol II HiChIP data for various distance stratification settings, using Hi-C data as background. Differential APA scores (Δscore) represent the difference in APA scores between the control and Auxin (i.e., CTCF depletion) backgrounds. (B) Log2 fold change (WT divided by STAG2-KD) in normalized Hi-C contact counts for upregulated and downregulated differential HiChIP loops for different settings of DiffHiChIP as well as for HiCDC+. We used IKAROS H3K27ac HiChIP data comparing WT to IKDN. (C) Recovery of reference differential Hi-C loops by DiffHiChIP for different distance stratification settings and statistical tests for the dataset in (A). *x* axis shows the top-k number of differential loops called for each method, and *y* axis shows the fraction of recovered differential Hi-C loops (reference data; *n* indicates their number). (D) Differential loops lost/weakened upon CTCF depletion (Auxin) in HCT116 that linked the gene *IER5L* and the ∼400 kb downstream super enhancer (marked 1). This loop was detected as differential by only edgeR glmQLFTest for the A + IHW setting. (E) Three differential loops (marked 1, 2, and 3; distance ∼250 kb) between WT and IKDN conditions for the IKAROS H3K27ac HiChIP data between *Jchain* and *Dck* genes and their detection by different settings of DiffHiChIP and HiCDC+ represented in a tabular format.

Availability of matched Hi-C data for the benchmarking studies prompted us to assess whether DiffHiChIP loops are also supported by differences in Hi-C signal between compared conditions. First, we performed differential APA analysis. For the loops downregulated upon CTCF depletion in HCT116 cells, we see strong differential APA patterns for A + BH, A + IHW, and A + D for all different edgeR settings (Figure 3A). DESeq2 failed to report a sufficient number of loops, and HiCDC+ reported none, to interpret any downstream analysis for the HCT116 data (Figures 2A and 3A). Similar analysis for differential loops from all three IKAROS HiChIP experiments showed differential APA enrichment across all edgeR settings combined with A + BH, A + IHW, or A + D with H3K27ac HiChIP data showing the strongest enrichment scores (Figures S3A–S3C). Next, for individual sets of differential loops detected from HiChIP data, we computed the log2 fold change of respective Hi-C contact counts between the compared conditions. Differences in Hi-C signal supported loss/decrease of looping for HiChIP differential loops detected by DiffHiChIP for the IKAROS and HCT116 datasets (Figures 3B, S3D–S3G, and S3H). We note that DESeq2-reported loops showed higher differences than those from edgeR settings only for the IKAROS H3K27ac HiChIP data (Figure 3B). Similarly, HiCDC+ showed higher differences than the edgeR settings for the Melanoma, IKAROS CTCF, and SMC1 HiChIP datasets, likely due to the method’s higher stringency (Figures S3F–S3H). Interestingly, the Hi-C fold change distributions were centered either near (Melanoma; Figure S3F) or at zero (HaCaT; Figure S3E) for two datasets, unlike IKAROS and HCT116 data, suggesting that the differences in HiChIP signal were mainly related to changes in the underlying 1D signal or “loop visibility” rather than true changes in 3D organization for HaCaT and Melanoma data (Data S2). However, APA using HiChIP background supported the differential loop calls for these two datasets (Figures S2C–S2H) highlighting the difficulty of distinguishing true loop changes solely from differential HiChIP analysis. Lastly, to evaluate the support of DiffHiChIP loops in Hi-C data, we defined a stringent set of reference differential Hi-C loops (or contact enrichments) by applying FitHiC2^36^ on the respective Hi-C datasets followed by a simple fold change criterion for filtering (STAR Methods). The A + IHW and A + D settings particularly with edgeR glmQLFTest and glmLRT models, respectively, recovered higher fraction of reference Hi-C loops for IKAROS and HCT116 datasets (Figures 3C, S4A, and S4D–S4F) whereas, for HaCaT and melanoma datasets, edgeR glmLRT and exactTest performed similar and better than the glmQLFTest model (Figures S4B and S4C). Overall, adjustment of *p* values using either IHW (A + IHW) or custom distance stratification (A + D) produced stronger Hi-C support compared to Benjamini-Hochberg (A + BH) correction. The underlying choice of statistical test mattered with A + IHW with glmQLFTest and A + D with glmLRT reaching highest levels of recovery in most cases (Figure S4). As previously discussed, A + IHW performed better than A + D in recovering longer-range loops, and Hi-C data supported these A + IHW exclusive loops (Figures S1 and S3).

#### Analysis of example loci for detecting long-range differential loops by distance stratification

To further compare different approaches in terms of their recovery of differences in long-range signals, we considered specific example loci analyzed in detail previously.^14^^,^^40^ For HCT116 data, we focused on differential loops between *IER5L* and a ∼400 kb downstream superenhancer (Figure 3D). Neither DESeq2 nor edgeR in A + H or A + D settings, nor HiCDC+, detected the ∼400 kb loop downregulated upon CTCF depletion whereas A + IHW with glmQLFTest reported this loop as differential (Figure 3D). For the same dataset, we also looked at another loop connecting *MYC* and a ∼1.9 Mb downstream superenhancer near the gene *GSDMC*, which was also detected as differential only by A + IHW with glmQLFTest (Figure S4G). For IKAROS H3K27ac HiChIP data, we focused on the *Jchain* locus, where we reported loss of >250 kb loops in our previous work.^40^ Consistent with higher recovery in genome-wide results (Figure 3C), A + IHW with glmQLFTest and A + D with glmLRT were the two combinations that captured differences in all three of the indicated loops whereas other settings missed either one or two of them (Figure 3E). These results support the aforementioned genome-wide observations in terms of A + IHW’s increased sensitivity for capturing *bona fide* long-range differences.

### edgeR with GLMs provides higher recall for differential loop calling

**Figure 4:**
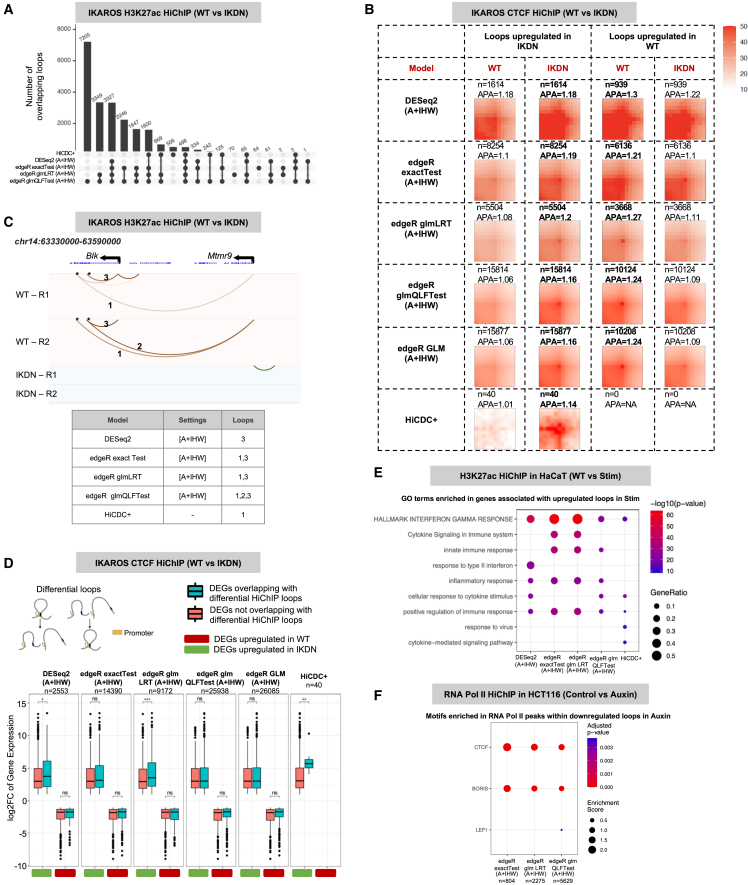
edgeR with GLM setting provides higher recall for differential loop calling (A) Overlap of differential loops between DESeq2 and various edgeR settings for the complete background with IHW-corrected FDR (setting A + IHW) for IKAROS H3K27ac HiChIP data. (B) APA plots for IKAROS CTCF HiChIP data between WT and IKDN conditions, for differential loops from DESeq2 and different edgeR settings. Values in bold denote expectation of higher APAs among the two conditions (i.e., upregulated loops in those conditions). (C) Three differential loops for *Blk* locus lost/weakened upon loss of IKAROS function and their tabulation, similar to (D). Loops 1 and 2 have ∼170 kb distance. (D) Enrichment of magnitude of gene expression change (log2 fold change) for differential genes segregated with respect to their overlap with DiffHiChIP loops from different settings for the IKAROS CTCF HiChIP dataset. Model (top right) of differential loops that overlap a gene promoter in at least one anchor. Significance was calculated using a Wilcoxon test (two-sided). ∗*p* <= 0.05; ∗∗*p* <=0.01; ∗∗∗*p* <=0.001; ∗∗∗∗*p* <=0.0001; ns, not significant. (E) Gene ontology and pathway enrichments for genes associated with upregulated H3K27ac loops after IFN-γ stimulation of HaCaT cells. Statistical significance (−log10*p* value) is shown by color scale, and gene ratio by circle size. (F) TF-binding motif enrichment within RNA-Pol-II ChIP-seq peaks overlapping downregulated loop anchors in Auxin condition. DESeq2 and HiCDC+ are not shown due to the limited number of ChIP-seq peaks overlapping differential loops detected (*n* = 40 and *n* = 0, respectively). Statistical significance is shown by color scale, and enrichment score by circle size. Schematic in (D) was created with Biorender.com/.

We next assessed DESeq2 and various statistical tests from edgeR (exactTest, glmLRT, and glmQLFTest) for differential loop calling when coupled with the A + IHW setting. DESeq2 with IHW and HiCDC+ reported a lower number of differential loops, most of which were covered by edgeR glm settings with IHW for all datasets (Figures 4A and S5A–S5F). Among the two GLMs, glmQLFTest reported a much higher number of differential loops in most cases (4 out of 6) compared to glmLRT. For the other two cases (HaCaT and Melanoma), glmLRT and edgeR exactTest reported highly overlapping differential calls that are missed by the glmQLFTest (Figures S5B and S5C). This prompted us to define another set of differential loops named edgeR GLM to denote the union of loops from glmLRT and glmQLFTest. Although APA between these GLMs alongside DESeq2 and edgeR exactTest did not reveal any striking difference in their APA scores, loops from DESeq2 showed stronger patterns in the bottom-left portion (i.e., the area that remains between the two anchors) highlighting the dominance of shorter-range loops among those reported by DESeq2 (Figures 4B and S6). Consistent with this, when we considered the ∼250 kb *Blk* locus with short- and long-range loop differences between WT and IKDN in H3K27ac HiChIP data, we observed that, out of three highlighted loops, DESeq2 reported only the short-range one as differential while edgeR models, in particular glmQLFTest, reported both the short- and long-range loop(s) as differential (Figure 4C). HiCDC+, on the other hand, only captured one long-range loop out of three highlighted as differential. Together with the three other examples discussed earlier (Figures 3D, 3E, and S4G), these results highlight the importance of using edgeR glm-based models for increased sensitivity of capturing differences spanning different distance ranges.

Next, we assessed the recovery of reference Hi-C loops or contact enrichments by different models. edgeR glmQLFTest settings recovered higher overall fraction of Hi-C loops for 4 out of 6 datasets (same 4 as earlier), but glmLRT settings led to a better ranking of significance evidenced by higher recovery at an equal number of differences reported (i.e., same value on the *x* axis) (Figures S4A–S4F). The edgeR exactTest and glmLRT models performed similarly for the remaining two datasets where glmQLFTest reported a low number (Figure S4B) or no differential loops (Figure S4C). HiCDC+ reported very low recovery across all conditions (Figures S4A–S4F).

We then evaluated whether the higher recall by some of the methods compared to the others comes at the expense of introducing more false positives. To assess this, we used our previously published HiChIP data from naive CD4^+^ T cells of multiple donors (*n* = 6). We artificially created two random partitions of 3 donors each (60 distinct combinations) and performed differential HiChIP loop analyses where any detected differential loop (aside from some genotype or sex-based differences) would represent false discoveries (Figure S5G). Across these comparisons, DESeq2, edgeR-based models, and HiCDC+ identified either zero or a very small number of differential loops, showing no large differences in FDRs across these models (Figure S5H).

### Downstream functional assessment of reported differential loops

Previous work by and us and others have shown that differences in regulatory HiChIP contacts are associated with larger changes in the expression of genes at the loops anchors.^11^ Thus, we assessed the association between fold changes of differentially expressed genes (DEGs) and presence of differential HiChIP loops from DiffHiChIP across different settings (STAR Methods). Our results indicate that, in many cases, there is a statistically significant difference between the fold changes of DEGs overlapping with differential HiChIP loops compared to those DEGs that do not (Figures 4D and S7). Looking at differences across methods, this difference was generally more prominent for DESeq2, edgeR glmLRT, and HiCDC+ differential loops although the results highly varied across datasets and across different pull-down targets for HiChIP (Figures 4D and S7).

We also have previously assessed the functional relevance of genes overlapping differential loops identified by edgeR glmQLFTest in the IKAROS dataset, where genes associated with downregulated loops after loss of IKAROS were enriched in pathways supporting B cell differentiation.^40^ We repeated similar analysis for the other HiChIP data analyzed in this work. For the HaCaT dataset, which compares unstimulated and IFN-γ-stimulated keratinocytes, we observed that genes associated with upregulated loops after stimulation were significantly enriched for terms related to IFN-γ response, inflammation, and positive regulation of immune response, consistently across DESeq2, edgeR models, and HiCDC+ (Figure 4E), although the enrichment for a number of related functional terms were method specific (e.g., cytokine signaling). Further analysis using transcription factor motif enrichment also highlighted the biological relevance of DiffHiChIP loops. For HCT116 RNA-Pol-II HiChIP data, which compares WT and CTCF-depleted cells, we observed that loops lost upon CTCF depletion were enriched for looping-related transcription factors such as CTCF and BORIS/CTCFL, across all the edgeR settings (Figure 4F) whereas DESEq2 and HiCDC+ did not lead to sufficient number of differential loops to carry out this analysis. For the IKAROS H3K27ac HiChIP data, we observed that H3K27ac ChIP-seq peaks within upregulated loop anchors in IKDN were enriched with transcription factors including TEAD, which has been shown to be directly repressed by IKAROS.^40^^,^^41^ Other transcription factors binding to motifs similar to IKAROS, such as NFAT, Esrrb, and AR, were also enriched in some edgeR settings but not the others (Figure S5I). DESeq2 and HiCDC+ differential loops were not significantly enriched for any motif for this IKAROS H3K27ac HiChIP data. Additionally, in our previous study,^40^ we have shown that IKAROS peaks within a downregulated H3K27ac loop identified by EdgeR showed strong enrichment of B cell lineage transcription factors, such as E2A and EBF1, at IKAROS-binding sites at a subset of sites. Together, these findings show the utility of edgeR GLMs in highlighting potentially important differential loops harboring the regulatory TFs.

### Comparison of different background estimation options

All the results so far used the complete background (A), basically union of HiChIP contacts (significant or not) across all input samples, as the background for underlying DESeq2 or edgeR settings. Previous studies such as HiCDC+^24^ and our previous work FitHiChIP,^23^ on the other hand, used a filtered subset of chromatin contacts to infer a background by considering only the contacts having FDR < *t* (user-defined threshold) in at least one input sample. FitHiChIP^23^ used *t* = 0.01 while HiCDC+ employed a more lenient *t* = 0.1. This approach of performing background estimation from only strong contacts (or loops) eliminates non-significant contacts with respect to their genomic distance-stratified background and can potentially reduce false-positive discoveries in the differential analysis. We implemented a similar filtered background estimation for DiffHiChIP coupled with two different distance stratification methods: (1) filtered background with IHW (model F + IHW) and (2) filtered background with distance stratification (model F + D).

**Figure 5:**
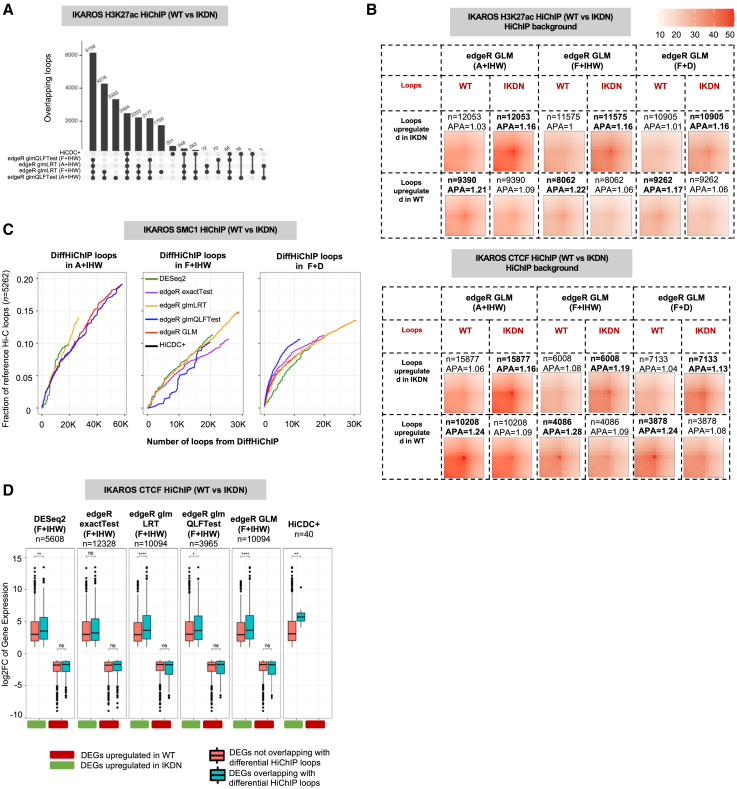
Assessment of background estimation on differential loop detection (A) Overlap of differential loops for various edgeR GLM settings between the complete (A + IHW) and filtered (F + IHW) background settings, and for HiCDC+, for the IKAROS H3K27ac HiChIP data. (B) APA plots for edgeR GLM setting (union of LRT and QLFTest) and for either A + IHW or F + IHW settings, with respect to IKAROS H3K27ac (top) and IKAROS CTCF (bottom) HiChIP datasets. Values in bold denote expectation of higher APAs among the two conditions (i.e., upregulated loops in that conditions). (C) Comparison between the complete (A) and filtered (F) backgrounds (A + IHW, F + IHW, and F + D) with respect to their recovery of differential Hi-C loops by different settings of DiffHiChIP for IKAROS SMC1 HiChIP dataset. The symbol *n* indicates the number of reference Hi-C loops. HiCDC+ tracks are not visible due to the limited number of differential loops detected (*n* = 43). (D) Enrichment of magnitude of gene expression change (log2 fold change) for differential genes segregated as was plotted in Figure 4D. Significance was calculated using a Wilcoxon test (two-sided). ∗*p* <= 0.05; ∗∗*p* <= 0.01; ∗∗∗*p* <= 0.001; ∗∗∗∗*p* <= 0.0001; ns, not significant.

First, we compared the complete (A + IHW) and filtered (F + IHW) backgrounds for IHW. For the HCT116 and IKAROS HiChIP datasets, loops from the setting A + IHW mostly included the loops from the setting F + IHW with ∼1.5–3 times more loops except the HaCaT and Melanoma datasets (Figures 5A and S8A–S8F). However, the F + IHW setting did not lead to any noticeable improvement in APA enrichment scores neither for IKAROS nor for any other dataset (Figures 5B, S10, and S11), even though for CTCF HiChIP data the F + IHW setting had ∼60% reduction in differential loops compared to A + IHW (Figure 5B). Considering the recovery of differential Hi-C loops, we mainly observed a decrease in overall recovery by F + IHW with some exceptions such as when the edgeR glmLRT model is used (Figures 5C and S8G–S8L). We also assessed the fold change of DEGs when they overlap with differential loops from F + IHW setting (Figures 5D and S9) and compared it to A + IHW but did not observe any noticeable or generalizable pattern of higher enrichment between A + IHW and F + IHW (Figure S7 vs. S9).

Comparing between IHW and distance stratification (D) using the filtered background (F + IHW and F + D) across all edgeR and DESeq2 settings and all datasets, F + IHW led to slightly higher APA scores for most cases, but this was not always the case and the differences were minimal (Figures 5B, S10, and S11). The F + IHW setting, particularly using edgeR models, recovered a higher fraction of reference Hi-C loops than the F + D setting in some datasets and showed comparable performance for the remaining (Figures 5C and S8G–S8L).

In terms of computational cost, the filtered background reduced runtime by around 2-fold compared to the complete background used with DiffHiChIP. After this reduction, the runtime of DiffHiChIP was very similar to that of HiCDC+. However, for memory usage, HiCDC+ was substantially more efficient compared to all different settings of DiffHiChIP, which were quite similar to one another (Figure S5J). Overall, resource utilization did not create a significant burden with runtimes up to 2 h and peak memory usage less than 50 Gb.

Overall, these results suggested limited utility of filtered background in terms of decreasing potential false-positive calls and modest gains in terms of resource utilization. Although they reported a similar number of differential loops, filtered background coupled with IHW correction generally performed better than distance stratification (F + D), especially in recovering differential Hi-C loops.

### DESeq2 sensitivity increases with higher number of replicates

**Figure 6:**
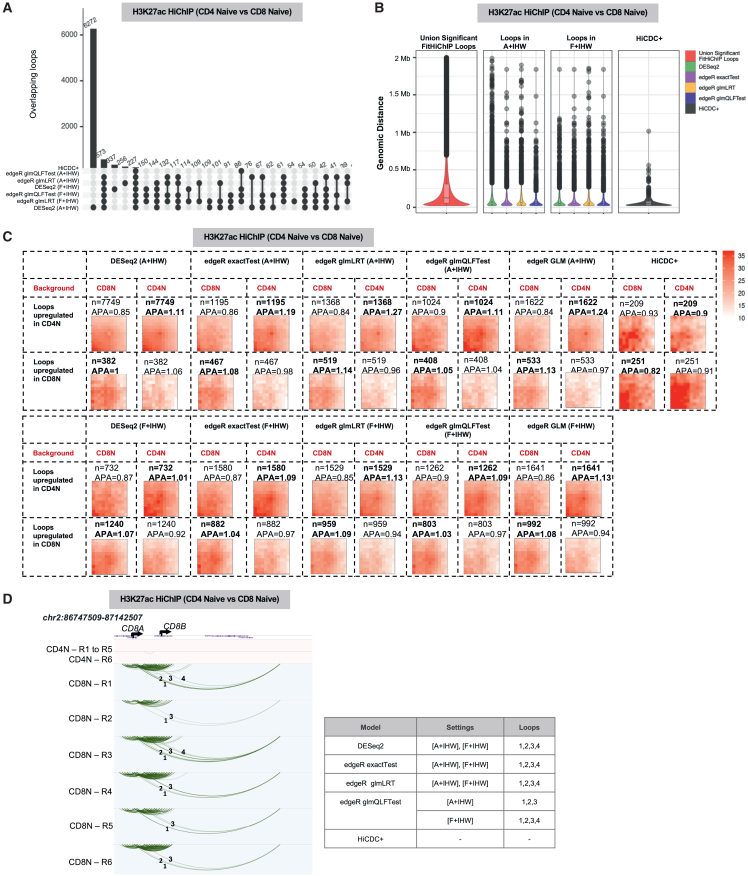
Impact of higher number of replicates on differential loop detection (A) Overlap of differential loops between naive CD4 and naive CD8 cell types for various settings of edgeR DESeq2 and for HiCDC+. (B) Genomic distance distributions for all A + IHW or F + IHW differential loops, together with the union of significant FitHiChIP loops of all replicates. (C) APA plots for DESeq2 and different edgeR settings and for the models A + IHW and F + IHW. Values in bold denote expectation of higher APAs among the two conditions (i.e., upregulated loops in that conditions). (D) Differential loops between naive CD4 and naive CD8 in the *CD8A* locus for different methods. Four CD8^+^ T cell-specific long-range loops involving the genes *CD8A* and *CD8B* are indicated by numbers.

Most of the HiChIP datasets in reference studies (and the results used so far) have very few (either 1 or 2) replicates per condition. To assess various settings of DiffHiChIP, we next used our previously published HiChIP data^11^ for naive CD4 and naive CD8 cell types each with six different donor samples as “replicates.” Such a higher number of replicates considerably increased the number of differential loops reported by DESeq2 (A + IHW) with ∼5 times more differential loops compared to various edgeR settings (Figure 6A) with a similar genomic distance distribution (Figure 6B). These results suggest that the output of DESeq2 is highly dependent on the number of replicates, an observation previously reported with respect to differential analysis of RNA-seq data.^42^ When the DESeq2 and edgeR results are compared with respect to different background choices, edgeR models reported a higher number of loops and lower APA enrichment in the filtered (F + IHW) setting compared to A + IHW (Figure 6C). Lack of any visible differential enrichment in the upregulated loops for most of the DESeq2 results together with high sensitivity to background choice suggests a large number of false positives (Figure 6C). When we specifically looked at differential loops involving the loci containing *CD8A* and *CD8B* (markers of CD8^+^ T cells), these were detected similarly by most edgeR settings and DESeq2 (Figure 6D) as upregulated in CD8^+^ T cells as expected. Further comparative analysis of different datasets with large numbers of replicates (>3) is needed to fully characterize these trends.

## Discussion

Although there are methods developed by us and others for identifying differential HiChIP loops,^23^^,^^24^^,^^25^ as well as for Hi-C and PCHi-C data,^19^^,^^20^^,^^21^^,^^22^ metrics to compare their results and studies that do this systematically across distinct datasets are lacking. Also, since the difference in HiChIP looping may be due to the changes in either chromatin folding or the underlying 1D (ChIP-seq) distribution, this assessment is even harder for HiChIP data. To date, no study has benchmarked differential HiChIP loop callers and assessed the impact of different distance stratification methods, background estimation, and statistical tests employed on their performance. Here, we present the first such large-scale benchmarking study while introducing new approaches (e.g., distance stratification) and creating a codebase that implements and makes available all the evaluated approaches for differential HiChIP loop calling.

DiffHiChIP is a comprehensive framework that incorporates reference count-based models DESeq2 and edgeR exactTest using either complete or pre-filtered backgrounds, includes edgeR-based GLMs, and simultaneously supports distance stratification by IHW and custom implementation. GLM-based regression coupled with LRT or QLFTest is expected to model the higher dispersion of HiChIP contacts better, while IHW-adjusted *p* values model the distance decay of chromatin contacts. DiffHiChIP is the first approach incorporating edgeR GLMs for differential HiChIP analysis motivated by their application in differential Hi-C loop calling (diffHiC^43^) and in single-cell RNA-seq studies modeling differential abundance.^33^^,^^34^

Although DESeq2 also computes regression by GLM, it estimates gene-wise dispersions and uses the Wald test for significance estimation. The GLMs in edgeR support both common and gene-wise dispersions, and the LRT or QLFTest are more reliable for modeling non-linear decay of HiChIP contacts with higher dispersion compared to 1D RNA-seq or ChIP-seq datasets. We note that these GLMs in DESeq2 or edgeR are, however, applicable when both input conditions have at least two replicates. When only a single replicate is available, DiffHiChIP defaults to applying the edgeR exactTest setting.

Our results show that classical BH-adjusted *p* values miss out on the differences of long-range chromatin interactions due to their lower contact counts, while the IHW or custom distance stratification techniques better recover them by modeling their distance decay. The IHW correction particularly performs well in capturing differences in long-range loops evidenced by multiple lines (different metrics) of support for such differences. For IHW, we did not use genomic distance as the covariate (as suggested by Gorkin et al.^38^) since the *baseMean* or *logCPM* values adequately represent the distance decay of chromatin contacts.

A higher number of replicates (>3 per condition) increases the number of detections by DESeq2, a phenomenon highlighted in a previous study,^42^, which also suggested using simple Wilcoxon rank-sum tests for count-based RNA-seq datasets with a high number of replicates. However, from our analysis, it was not clear what fraction of the additional discoveries by DESeq2 were *bona fide* changes and not false positives. Given that HiChIP datasets usually have lower number of replicates (1 or 2) per category, edgeR models with IHW are potentially more preferable given their applicability to most cases and superior performance according to multiple different metrics.

DiffHiChIP also incorporates options from existing differential HiChIP loop callers, including diffloop and FitHiChIP, which employ edgeR exactTest with pre-filtered background, with diffloop additionally filtering out loops detected in a single sample. Another published method HiCDC+ employs DESeq2 with library size factors estimated separately for individual distance bins (default 10 kb). Our results show that HiCDC+ and DESeq2 recover a lower fraction of reference Hi-C loops and do not detect differential loops in various example loci, compared to the edgeR GLMs, suggesting lower sensitivity. Similar to our earlier work FitHiChIP,^23^ DiffHiChIP can identify the differential and non-differential loop anchors between conditions by applying edgeR onto the 1D ChIP-seq (if additionally provided as an input) coverage between conditions. This way FitHiChIP characterizes the differential interactions involving no differences or only small differences in the underlying 1D signal in each anchor allowing for segregating differential loops that can be explained by visibility differences from those that largely are due to changes in 3D chromatin organization (Data S2).

Overall, DiffHiChIP is a comprehensive framework for differential HiChIP analysis that combines multiple approaches used to date and introduces new options to improve capture of differences in long-range loops alongside shorter-range loops. With the ever-increasing number of HiChIP datasets generated to compare multiple conditions/perturbations across different biological systems, we believe the presented results will be of high interest to the field, and the developed framework will be highly utilized. We make our documented source code and package available on GitHub and all of the produced data files (differential loop calls across all datasets and all DiffHiChIP settings discussed in this work) through a web server at https://ay-lab-tools.lji.org/DiffHiChIP/.

### Limitations of the study

While DiffHiChIP provides a comprehensive framework for detecting differential chromatin loops, several considerations should be kept in mind when interpreting its results. Differential HiChIP loops can reflect either changes in 3D chromatin folding or differences in the underlying 1D ChIP-seq signal, and DiffHiChIP can only distinguish between these sources when matched ChIP-seq data are available. Additionally, GLM-based models in edgeR require at least two replicates per condition, limiting their applicability in datasets without replicates. Lastly, it is important to note that no single evaluation metric we employed here is fully informative of superior performance on its own and has to be represented in the context of all other metrics. For instance, APA scores could be maximized by capturing a minimal number of loops with the largest differences, but this will lead to low sensitivity. It is also possible that higher APA for one method compared to the other can come at the cost of the first method missing out on longer-range loops. Better recovery of differential Hi-C (or reference) loops by one particular method may be mostly a result of a much larger number of differential calls that may be related to lower specificity. Thus, performance must be interpreted in the context of multiple complementary metrics, even if this adds complexity to the evaluation.

## Resource availability

### Lead contact

Requests for further information and resources should be directed to and will be fulfilled by the lead contact, Ferhat Ay (ferhatay@lji.org).

### Materials availability

This study did not generate new unique reagents.

### Data and code availability

• This paper analyzes existing publicly available data available at NCBI GEO. DOIs or accession numbers for these datasets are listed in the key resources table.
• Code for DiffHiChIP is publicly available in the GitHub repository https://github.com/ay-lab/DiffHiChIP and a stable copy as of October 2025 is uploaded to the Zenodo repository https://zenodo.org/records/17410330.
• Differential loop results and WashU browser tracks for all datasets and DiffHiChIP settings are accessible via our web server https://ay-lab-tools.lji.org/DiffHiChIP/ as well as from the Zenodo repository https://zenodo.org/records/17410330.

## Acknowledgments

We thank the members of the Ay and Georgopoulos labs for their valuable support. We thank Laura Hinojosa for help with illustrations. This work was funded by 10.13039/100000002NIH grants R35-GM128938 (F.A.) and R01-HL140622 (K.G. and F.A.).

## Author contributions

Conceptualization and algorithm development, S.B., D.S.F., and F.A.; data analysis, S.B. and D.S.F.; writing – original draft & writing – review & editing, S.B., D.S.F., F.A., and K.G.; supervision and funding acquisition, F.A. and K.G. All authors read and approved the manuscript.

## Declaration of interests

The authors declare no competing interests.

## Declaration of generative AI and AI-assisted technologies in the writing process

During the preparation of this work, the authors used ChatGPT in order to improve the readability and language of the manuscript. After using this tool, the authors carefully reviewed and edited the content as needed and take full responsibility for the content of the published article.

## STAR★Methods

### Key resources table

**Table:** 

REAGENT or RESOURCE	SOURCE	IDENTIFIER
**Deposited data**		

RNA-Pol-II HiChIP data in WT and CTCF depleted HCT116 cells	Lee et al.^14^	GEO: GSE179545
H3K27ac HiChIP data in unstimulated and IFN-γ stimulated keratinocytes	Shi et al.^15^	GEO: GSE151193
H3K27ac HiChIP in WT and STAG2 mutant M14 cells	Chu et al.^13^	GEO: GSE156773
H3K27ac, CTCF and SMC1 HiChIP in WT and IKAROS mutant large pre-B cells	Hu et al.^40^	GEO: GSE232490
H3K27ac HiChIP data in naive CD4^+^ and CD8^+^ T cells	Chandra et al.^11^	dbGaP: phs001703.v3.p1
Source code and output files from presented analysis	This paper	Zenodo: https://zenodo.org/records/17410330

**Software and algorithms**		

DiffHiChIP	This paper; GitHub	https://github.com/ay-lab/DiffHiChIP
FitHiC v.2.0.7	Kaul et al.^36^	https://github.com/ay-lab/fithic
FitHiChIP v9.1	Bhattacharyya et al.^23^	https://ay-lab.github.io/FitHiChIP/html/index.html
GENOVA v1.0.0.9	van der Weide et al.^44^	https://github.com/robinweide/GENOVA
GenomicRanges v1.42.0	Lawrence et al.^45^	https://bioconductor.org/packages/release/bioc/html/GenomicRanges.html
Gene Ontology (GO) analysis	Zhou et al.^46^	https://metascape.org
HiCDC+ v1.14.0	Sahin et al.^24^	https://www.bioconductor.org/packages/release/bioc/html/HiCDCPlus.html
HiC-Pro v2.11.4	Servant et al.^47^	https://github.com/nservant/HiC-Pro
Homer v5.1	Heinz et al.^48^	http://homer.ucsd.edu/homer/
Juicebox v3.1.0	Durand et al.^49^	https://github.com/aidenlab/Juicebox
MACS2 v2.2.9.1	Zhang et al.^50^	https://github.com/macs3-project/MACS
ROSE v1.3.2	Whyte et al.^51^	https://github.com/stjude/ROSE
STAR v2.7.1	Dobin et al.^52^	https://github.com/alexdobin/STAR

### Method details

#### Overview of DiffHiChIP

##### Input data

DiffHiChIP is a comprehensive framework for calling differential loops primarily from HiChIP data. It employs loop calls derived for the input samples computed using either FitHiChIP^23^ or other HiChIP loop callers. We note that all input samples should be processed by the same chromatin loop caller employing the same parameters (resolution, distance thresholds, etc.). Complete list of all interactions for individual samples (whether they are loops with respect to some filtering by their significance values) along with their statistical significance values are provided as an input to DiffHiChIP.

##### Background loops

DiffHiChIP supports two different sets of background loops for the underlying DESeq2 or edgeR settings: 1) complete (A) background: using the union of chromatin interactions (nonzero contact counts) from all the input samples, and 2) filtered (F) background: using the union of loop calls with FDR < *t* for at least one input sample, where *t* is a user-defined FDR threshold with default 0.1 (similar to HiCDC+^24^).

##### Significance thresholds for differential analysis

After applying the DESeq2 or edgeR models for significance estimation, loops with adjusted *p*-values (from DESeq2 or edgeR models) < *f*, absolute log fold change > *l,* and statistically significant (FDR from the chromatin loop caller < *t*) in at least one input sample are returned as differential, where *f*, *l* and *t* are user-defined thresholds with default values of 0.05, 1 and 0.01, respectively.

##### Applying DESeq2

The design variable for DESeq2 is constructed using the input condition information. Reference functions from the DESeq2 Bioconductor package such as *DESeqDataSetFromMatrix*, *DESeq*, and *results* are employed.

##### Applying edgeR

DiffHiChIP incorporates edgeR supporting both exactTest and GLM models. Reference routines from the edgeR Bioconductor package are used, such as *estimateDisp* for estimating dispersions, *exactTest* for the exactTest model, *glmFit* and *glmLRT* for GLM with LRT model, and *glmQLFit* and *glmQLFTest* for modeling GLM with QLFTest.

##### IHW for distance stratification

DiffHiChIP supports applying independent hypothesis weighting (IHW) on the resulting *p*-values from DESeq2 or edgeR, by applying the routine *ihw* from the Bioconductor package IHW. The baseMean and logCPM values for each interaction are used as the covariates for DESEq2 and edgeR, respectively. The *alpha* parameter for *ihw* routine is kept the same as the user-defined significance threshold *f* for differential analysis (mentioned above).

##### Custom distance stratification

DiffHiChIP also provides a custom implementation of distance stratification of chromatin loops, to mitigate the distance decay bias. We adapted the equal occupancy binning described in our previous method FitHiChIP.^23^ If *N* is the number of locus pairs and *C* is the total number of contacts between them (sum of contact counts), then considering *M* bins (we considered *M* = 300 for the default distance range from 10 Kb to 3 Mb equally spaced by 10 Kb), each bin would have ∼ *C*/*M* contacts. We first sorted the interactions by their genomic distance values, assigned them into 10 Kb binning intervals according to their interaction distance values, and then constructed the equal occupancy bins such that each bin has at least *C*/*M* contacts. Each of these equal occupancy bins and their constituent interactions were then applied to the downstream DESeq2 or edgeR models for estimating the *p*-values. Finally, all the *p*-values from all the interactions across all equal occupancy bins are subjected to BH-correction. Note that when this distance stratification is employed, IHW is not used to avoid double correction.

##### Differential loops involving non-differential anchors

To identify differential HiChIP loops involving non-differential 1D anchors (with respect to the looping resolution such as 5Kb), we applied edgeR exactTest on the given ChIP-seq coverage information between conditions (if available for the specific conditions) and labeled the anchors with significant differences at 5% FDR as differential between conditions with respect to 1D ChIP-seq coverage. Differential HiChIP loops involving only the non-differential 1D anchors were regarded as differences largely explained by the true changes in 3D chromatin folding between conditions. ChIP-seq coverage (bedgraph) was derived from the respective alignment (bam) file using the *bamtobed* utility from bedtools. Here edgeR exactTest was employed since the input ChIP-seq data may not have replicates for the respective conditions. Note that HiChIP 1D coverage was not used for identifying the differential loop anchors.

#### Dataset description

We used the following HiChIP datasets to validate DiffHiChIP: 1) RNA-Pol-II HiChIP data from HCT116 colorectal cancer cells^14^ (available from Gene Expression Omnibus or GEO repository under the accession number GSE179545) in two conditions: untreated (or control) and treated with Auxin to induce a degron system on CTCF. This dataset has Hi-C, RNA-seq and RNA-Pol-II ChIP-seq data for the corresponding conditions and replicates. 2) H3K27ac HiChIP data in unstimulated and IFN-γ stimulated HaCaT cells^15^ (GEO: GSE151193). Accompanying RNA-seq and Hi-C data for the corresponding conditions are also provided. 3) H3K27ac HiChIP data from M14 melanoma cells expressing doxycycline-inducible shRNA targeting STAG2 treated with (STAG2-KD) or without (WT) doxycycline^13^ (GEO: GSE156773). 4) H3K27ac, CTCF and SMC1 HiChIP from mouse large pre-B cells in two conditions: wild-type (WT) and IKAROS mutant (IKDN), which correspond to an *in vivo* deletion of *Ikzf1* exon 5 encoding the IKAROS DNA-binding domain^40^ (GEO: GSE232490). This dataset has matching Hi-C, RNA-seq and H3K27ac, CTCF and SMC1 ChIP-seq data. 5) H3K27ac HiChIP data in two immune cell types naive CD4 and naive CD8 prevalent in human peripheral blood mononuclear cells (PBMCs) from six donors.^11^ This data is available through the database of Genotypes and Phenotypes (dbGaP) under the accession number phs001703.v3.p1. We note that datasets 1 to 4 have 2 replicates per condition, while the dataset 5 has 6 replicates (different donors) per condition.

#### HiChIP data processing and loop calling

HiChIP paired-end reads were aligned to the human hg38 or mouse mm10 (for the IKAROS dataset) genome assembly using the HiC-Pro^47^ pipeline (version 2.11.4). Default settings were used to remove duplicate reads, assign reads to restriction fragments, filter for valid pairs, and generate raw and ICE normalized interaction matrices at a range of resolutions. For visualization, valid pairs were converted to.hic files using the script *hicpro2juicebox.sh* from Juicebox v3.1.0.^49^ ChIP-seq peak calling was performed using MACS2^50^ (version 2.2.9.1) with input chromatin as control and with a q-value cutoff of 0.05. FitHiChIP^23^ (version 9.1) was used to identify statistically significant HiChIP interactions from individual samples, employing the ChIP-seq data generated independently from the same study/conditions as the HiChIP libraries. HiChIP interactions were called using peak-to-all background setting (UseP2PBackgrnd = 0), 5Kb bin size, and distance range between 10Kb and 3Mb. For the SMC1 and CTCF HiChIP datasets in,^40^ only peak-to-peak interactions were assessed for significance, i.e., when both anchors overlapped a ChIP-seq peak. For all other HiChIP datasets, peak-to-all interactions were considered, i.e., at least one anchor needed to overlap with a reference ChIP-seq peak.

#### Differential loop analysis using HiCDC+

To enable a direct comparison with DiffHiChIP, we performed differential loop calling using the *hicdcdiff* function from the HiCDCPlus R package (version 1.14.0). We set the *fitType* parameter to 'mean', and as input, we used the loops identified by FitHiChIP at a significance threshold of q-value <0.1. This configuration allowed us to assess the performance of HiCDC+ under the same input data and similar filtering conditions as DiffHiChIP, facilitating a consistent comparison between the two differential loop detection methods.

#### Aggregate peak analysis

To show the average contact count distribution of loops and their surroundings, we performed an aggregate peak analysis (APA) of loop calls using the R package GENOVA v1.0.0.9.^44^ For aggregate signal assessment, we used either HiChIP or Hi-C contact maps binned at 10 kb resolution that were normalized by Knight-Ruiz (KR). We retrieved the normalized contact counts of a 100 kb × 100 kb region centered on each loop coordinate corresponding to a pair of loci on the same chromosome represented by coordinates: *i* and *j*. Without loss of generality, we can assume coordinate *i* < *j* and represent their genomic distance by d = d(*i*,*j*) = *i* – *j* for a given loop. The APA then plots the average contact count across all 100 kb × 100 kb with center pixel depicting (*i*,*j*), bottom left pixel corresponding to (*i*+50kb, *j*-50kb) and top right pixel denoting (*i*-50kb, *j*+50kb) with genomic distances of d, d-100kb and d+100kb, respectively. In alignment with the literature, only loops with a genomic distance greater than 130kb are considered in the APA analysis. The APA score displayed on top of each plot is the ratio between the central pixel value and the mean value of pixels 15–30 kb downstream of the upstream loci and 15–30 kb upstream of the downstream loci.

We computed APA scores separately for the loops upregulated in each condition and, for each set, we use the underlying contact map from each condition separately, thus, providing us with four different APA plots and scores. Let *S*_*XY*_ be the APA score for the loops upregulated in the condition *X* and with respect to the background set of HiChIP contacts from the condition *Y*. A set of differential loops between two input conditions *A* and *B* should ideally satisfy the condition *S*_*AA*_ *>= S*_*AB*_ and *S*_*BB*_ *>= S*_*BA*_, that is, differential loops upregulated in a given condition should also be more enriched in the respective background set of loops. We denote *d*_*AB*_ = (*S*_*AA*_ - *S*_*AB*_) as the *differential APA score* for the loops upregulated in the condition *A*. Thus, higher values of *S*_*AA*_ and *d*_*AB*_ indicate higher enrichment of loops in the condition *A*.

#### Calling superenhancers from ChIP-seq peaks

For the Auxin dataset (GEO: GSE179545) we identified superenhancers with the Rank Ordering of Super-Enhancers (ROSE) v1.3.2 algorithm^51^ using the H3K27ac ChIP-seq peaks and the default stitching size of 12.5 kb.

#### Overlap of HiChIP/Hi-C loops between different models

To compare HiChIP loops between different settings of DiffHiChIP, we used the exact overlap strategy (i.e., identical anchors). To check the recovery of reference differential Hi-C loops by the DiffHiChIP output loops, we employed a slack of 5Kb similar to our previous work,^23^ that is, declared two different loops overlapping if their respective anchors were within 5Kb (one bin size) of each other.

#### Recovery of differential Hi-C loops by DiffHiChIP

Hi-C paired-end reads were aligned to the human hg38 or mouse mm10 (for the IKAROS dataset) genome assembly using the HiC-Pro v2.11.4^47^ pipeline as described under the HiChIP data processing section. FitHiC2^36^ was used with default parameters to identify statistically significant Hi-C interactions. We defined the reference differential Hi-C loops using EdgeR_exactTest and a criterion of log fold change >2 between the input conditions. We then computed the fraction of these reference differential Hi-C loops recovered by the differential HiChIP loops for different settings of DiffHiChIP, subject to increasing number of loop calls (decreasing stringency), and according to the above-mentioned criteria of loop overlap.

#### Differentially expressed genes and their overlap with differential loops

RNA-seq paired-end sequencing reads were aligned to the human hg38 or mouse mm10 (for the IKAROS dataset) genome assembly using STAR v2.7.1^52^ with default parameters. Normalization and differential gene expression analysis was performed using DESeq2 v1.40.2.^27^ Differentially expressed genes (DEG) were identified using an adjusted *p*-value cutoff of 0.05. A gene was considered to overlap with differential HiChIP loops if its transcription start site (TSS) was located within a differential loop anchor. The overlap of genomic regions was performed using the R package GenomicRanges v1.42.0.^45^ Metascape, a free gene annotation and analysis resource, was used for Gene Ontology (GO) analysis (https://metascape.org).^46^

#### Evaluation of the type I error rate

To assess the type I error rate of, we utilized the T cells dataset, which profiles naive CD4^+^ and CD8^+^ T cells obtained from six different human donors. For the purpose of this analysis, we focused on the CD4^+^ T cell samples. To simulate a null scenario in which no true differential looping is expected, we randomly assigned the CD4^+^ replicates into two artificial groups. This random assignment was repeated independently across 60 iterations. For each iteration, we applied our differential loop analysis to test for statistically significant differences in looping between the two artificial conditions. Significance was determined using a false discovery rate (FDR) threshold of 5%.

#### Motif enrichment analysis

We first overlapped the ChIP-seq peak summits specific to each condition with the anchors of the upregulated and downregulated loops. The overlapping ChIP-seq peak summits ±200 bp regions were employed for motif enrichment analysis using HOMER v5.1,^48^ with respect to the background set of complete ChIP-seq peaks, and using the parameters *“-size given -mask”.* Significant known motifs (adjusted *p*-value <0.01) were visualized and its enrichment was calculated as log2(% of target sequences/% of background sequences). DiffHiChIP settings with fewer than 100 ChIP-seq peaks overlapping differential loops were excluded from the analysis.

#### Statistics and reproducibility

R v4.0.1 was used for statistical analysis and plotting of data. The statistical significance between two groups was assessed using a two-sided Wilcoxon test.

## Supplemental information

Document S1. Figures S1–S11

Data S1. Lists the input HiChIP datasets, HiChIP statistics, and the number of FitHiChIP loops for these datasets, related to Figure 1 and STAR Methods

Data S2. Fraction of differential loops involving non-differential ChIP-seq signal at loop anchor with respect to underlying conditions, related to Figures 2–6 and STAR Methods

Document S2. Article plus supplemental information
